# Infographic: intravitreous aflibercept vs vitrectomy with panretinal photocoagulation for vitreous haemorrhage from proliferative diabetic retinopathy

**DOI:** 10.1038/s41433-021-01530-0

**Published:** 2021-05-26

**Authors:** Anna Song, Ali Ghareeb, Alexander Mehta, Salman Naveed Sadiq, Mohaimen Al-Zubaidy, Declan C Murphy, Islam Mostafa, Nikolaos Tzoumas, David H. W. Steel

**Affiliations:** Biosciences Institute, Newcastle University, Newcastle upon Tyne, UK

**Keywords:** Outcomes research, Retinal diseases, Drug therapy

**Fig. 1:**
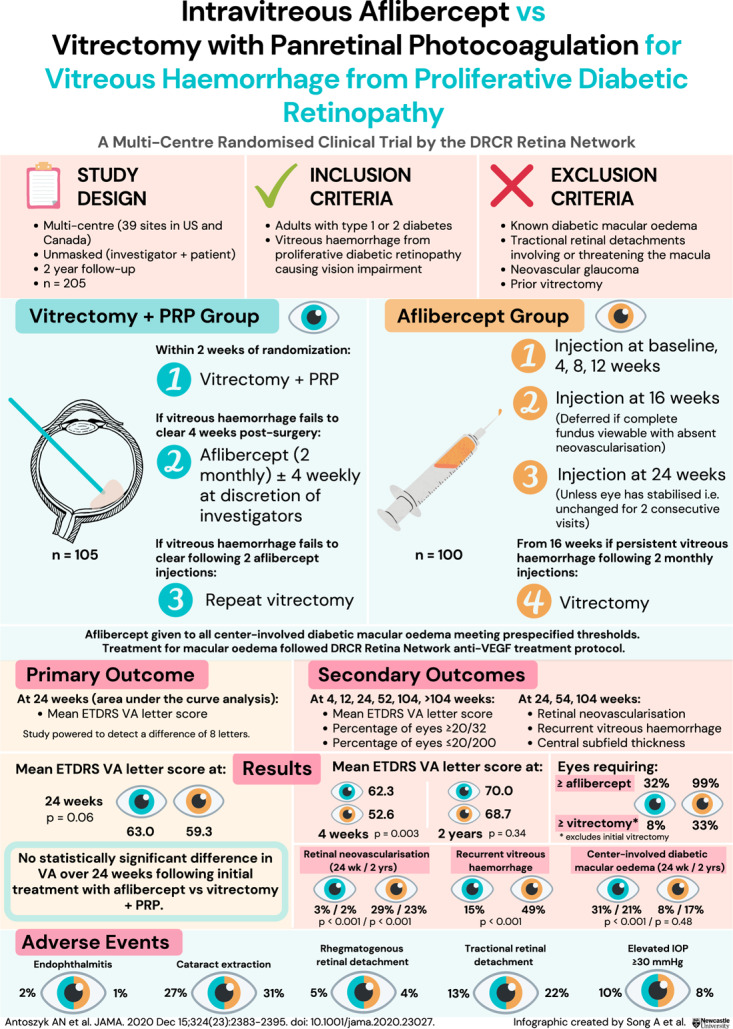
The intravitreous aflibercept vs vitrectomy with panretinal photocoagulation for vitreous haemorrhage from proliferative diabetic retinopathy randomised clinical trial by the DRCR Retina Network showed that there was no statistically significant difference in visual acuity at 24 weeks following initial treatment with aflibercept compared to vitrectomy with panretinal photocoagulation. PRP panretinal photocoagulation, DRCR Diabetic Retinopathy Clinical Research, ETDRS Early Treatment Diabetic Retinopathy Study, VA visual acuity.

**Reference to original study:** Antoszyk AN, Glassman AR, Beaulieu WT, Jampol LM, Jhaveri CD, Punjabi OS, et al.; DRCR Retina Network. Effect of Intravitreous Aflibercept vs Vitrectomy With Panretinal Photocoagulation on Visual Acuity in Patients With Vitreous Hemorrhage From Proliferative Diabetic Retinopathy: A Randomized Clinical Trial. JAMA. 2020 Dec 15;324(23):2383–2395.

